# Increased sLRP1 and decreased atrial natriuretic peptide plasma levels in newly diagnosed T2DM patients are normalized after optimization of glycemic control

**DOI:** 10.3389/fendo.2023.1236487

**Published:** 2023-08-10

**Authors:** Eduardo García, Pedro Gil, Inka Miñambres, Aleyda Benitez-Amaro, Claudia Rodríguez, Lene Claudi, Josep Julve, Sonia Benitez, Jose Luís Sánchez-Quesada, Jose Rives, Xavier Garcia-Moll, David Vilades, Antonio Perez, Vicenta Llorente-Cortes

**Affiliations:** ^1^ Institut de Recerca de l’Hospital de la santa Creu i Sant Pau, Sant Quintí, Barcelona, Spain; ^2^ Biomedical Research Institute Sant Pau (IIB Sant Pau), Sant Quintí, Barcelona, Spain; ^3^ Institute of Biomedical Research of Barcelona (IIBB), Spanish National Research Council (CSIC), Barcelona, Spain; ^4^ Universitat Autònoma de Barcelona, Barcelona, Spain; ^5^ Endocrinology and Nutrition Service, Hospital de la Santa Creu i Sant Pau, Sant Quintí, Barcelona, Spain; ^6^ Centro de Investigación Biomédica en Red de Diabetes y Enfermedades Metabólicas (CIBERDEM), Instituto de Salud Carlos III, Madrid, Spain; ^7^ Cardiology Department, Santa Creu i Sant Pau University Hospital, Barcelona, Spain; ^8^ Cardiac Imaging Unit, Cardiology Department, Santa Creu i Sant Pau University Hospital, Barcelona, Spain; ^9^ Centro de Investigación Biomédica en Red de Enfermedades Cardiovasculares (CIBERCV), Institute of Health Carlos III, Madrid, Spain

**Keywords:** sLRP1, ANP, metabolic control, T2DM, hypoglycemic treatments

## Abstract

**Background:**

Low-density lipoprotein receptor-related protein 1 (LRP1) negatively modulates circulating atrial natriuretic peptide (ANP) levels. Both molecules are involved in the regulation of cardiometabolism.

**Objectives:**

To evaluate soluble LRP1 (sLRP1) and ANP levels in people with newly diagnosed type 2 diabetes mellitus (T2DM) and determine the effects of metabolic optimization.

**Methods:**

This single-center longitudinal observational study recruited patients with newly diagnosed T2DM (*n* = 29, HbA1c > 8.5%), and 12 healthy control, age- and sex-matched volunteers. sLRP1 and ANP levels were measured by immunoassays at T2DM onset and at one year after optimization of glycemic control (HbA1c ≤ 6.5%).

**Results:**

T2DM had higher sLRP1 levels than the control group (p = 0.014) and lower ANP levels (p =0.002). At 12 months, 23 T2DM patients reached the target of HbA1c ≤ 6.5%. These patients significantly reduced sLRP1 and increased ANP levels. Patients who did not achieve HbA1c < 6.5% failed to normalize sLRP1 and ANP levels. There was an inverse correlation in the changes in sLRP1 and ANP (p = 0.031). The extent of sLRP1 changes over 12 months of metabolic control positively correlated with those of total cholesterol, LDL cholesterol, TG, TG/HDLc, and apolipoprotein B.

**Conclusions:**

Newly diagnosed T2DM patients have an increased sLRP1/ANP ratio, and increased sLRP1 and decreased ANP levels are normalized in the T2DM patients that reached an strict glycemic and metabolic control. sLRP1/ANP ratio could be a reliable marker of cardiometabolic function.

## Introduction

Patients with type 2 diabetes mellitus (T2DM) show higher incidence of heart failure and higher cardiovascular disease (CVD) mortality risk (1). Achieving optimal glycemic control is a clinical goal in persons with T2DM to reduce CVD risk, especially at early stages of T2DM (2, 3). However, new biomarkers connecting cardiovascular and metabolic alterations need to be developed for helping to prevent CVD in T2DM.

Low-density lipoprotein receptor-related protein 1 (LRP1) plays a crucial role in atherosclerosis progression (4, 5). Its soluble form (sLRP1) comprises the α chain (515 kDa) and a fragment of the β chain (55 kDa) of the cellular receptor. sLRP1 can be detected in the circulation after either constitutive or induced cleavage (6). Previous studies from our group have shown that atherogenic lipoproteins promote sLRP1 release from vascular smooth muscle cells of human atherosclerotic plaque explants, and that circulating levels of sLRP1 are associated with carotid atherosclerosis in familial hypercholesterolemic patients (7) and with carotid plaque inflammation measured by 18F-FDG PET in patients with a recent ischemic stroke (8). In line with this, we further demonstrated that circulating sLRP1 levels are also associated with coronary artery disease risk, independently of potential confounding factors (9).

LRP1 is overexpressed in advanced human atherosclerotic lesions enriched in lipids (10), in the vascular wall of hypercholesterolemic rabbits and pigs (5, 11, 12) and in myocardium of *in vivo* models of diabetic rats (13).

In the context of cardiometabolic diseases, epicardial fat expresses and releases an altered pattern of adipokines and other molecules that contribute to vascular and cardiac dysfunction (14, 15). As LRP1 is overexpressed in epicardial fat of T2DM patients (16), it could be hypothesized that dysfunctional epicardial fat contributes to increased circulating sLRP1 levels. Supporting this, sLRP1 levels directly correlate with epicardial fat volume in type 1 diabetes mellitus (T1DM) patients and in the general population (17, 18). Together, these results suggest that sLRP1 could be a dual biomarker of cardiac and metabolic alterations in T2DM.

Atrial natriuretic peptide (ANP) is secreted by cardiomyocytes (19), and its circulating concentration decrease under insulin resistance (20, 21). Our group has previously shown in a murine model that the circulating levels of ANP are modulated by cardiac LRP1 levels, and that the reduction of cardiomyocyte LRP1 levels increases the release of ANP that coordinately inhibits hepatic fatty acid (FA) synthesis and activates FA uptake and oxidation, limiting weight gain and enhancing whole-body energy consumption (22).

LRP1 levels in heart inversely modulate ANP levels (22), which is essential in the control of metabolism. In light of this, the sLRP1/ANP ratio could be used as an indicator that integrates information about metabolic and cardiovascular alterations. Our hypothesis is that the inverse relation between the circulating levels of sLRP1 and ANP in humans are favorably influenced by the optimization of the glycemic control. The aims of this study were i) to evaluate sLRP1 and ANP levels in newly diagnosed T2DM patients as compared to healthy controls, and ii) to determine the effect of achieving metabolic optimization on these circulating biomarkers.

## Methods

### Patients

This single-center, observational longitudinal study included 29 patients with T2DM (mean age 56 ± 9 years) who had been referred to the Endocrinology and Nutrition Department of Hospital de la Santa Creu i Sant Pau, Barcelona (Spain), as well as 12 age- and sex-matched control subjects. The study was approved by the Ethics Committee of the Hospital de Sant Pau (reference number of the protocol IIBSP-REL-2017-27, data of approval 07/26/2017). Written informed consent was obtained from all participants. This study was performed in full compliance with the Declaration of Helsinki. T2DM patients were newly diagnosed without previous hypoglycemic, lipid-lowering or anti-inflammatory drugs. Control group were normolipemic and normoglycemic subjects, with no personal or family history of premature coronary disease, major cardiovascular risk factors, or infectious or inflammatory disease.

All T2DM patients (n = 29) were studied at onset and at 12 months after treatment started. Patients were subjected to an intensive intervention carried out to achieve recommended targets of cardiovascular risk factors that includes diet, physical activity and pharmacotherapy, in accordance with clinical practice guidelines. Lipid profile, glucose, CRP and HbA1c levels were determined by routine procedures. All T2DM patients participated in a comprehensive diabetes self-management education program, which includes individualized instruction on nutrition, physical activity and optimizing metabolic control. According to our protocol for the management of severe hyperglycemia, the initial therapy included triple therapy with metformin, dipeptidyl peptidase inhibitors and basal insulin in 90% of patients. Basal insulin was suspended after 1–2 weeks, and non-insulin drugs were modified at the discretion of the responsible physician according to the characteristics of each patient.

### Immunoassays

Blood samples were obtained at disease onset and 12 months of treatment start by venipuncture in EDTA-containing or additive-free Vacutainer tubes to obtain plasma or serum, respectively. Tubes were centrifuged for 15 min at 1500*g* at room temperature for a maximum period of 30 min. The resulting plasma or serum was aliquoted and stored at –80 until use. sLRP1 levels were measured in plasma using an ELISA kit from Cloud-Clone corp (SEB010Hu), and ANP levels were measured in serum using an ELISA kit from LSBio kit (LS-F57269).

### Statistical analysis

In order to justify the validity of the kits used, we have revised the ELISA data in terms of sensitivity limits and reliability of results. The Intra-Assay CV for LRP1 ELISA Kit was <10% and for ANP ELISA Kit <6.9%. The coefficients of variation Inter-Assay was CV<12% for LRP1 ELISA kit and CV<8.7% for ANP ELISA Kit. These intra-assay and inter-assay coefficient of variation (CV) support the quality and feasibility of these assays to be used as evaluation tools. The power was between 0.78 and 0.96 with N of 29 patients and 8 controls for the variables sLRP1, ANP and sLRP1/ANP. The normality of numerical data distribution was verified using the Shapiro-Wilk test, and the homoscedasticity, with the Levene’s test. The categorical variables are presented as frequencies and percentages, and the quantitative variables are presented as mean ± SD. A bivariate analysis was used for paired data, and a Chi-square analysis was used for categorical data. Relationships between different Δparameters and Δbiomarkers were assessed using Spearman correlation analysis. To determine the possible confounding factors between the associations of different variables, a simple linear regression test was used. Due to the small sample size, the analysis was validated using a non-parametric approach. All statistical analyses were performed with SPSS software, version 27. A two-sided *p*-value < 0.05 was considered statistically significant.

## Results

### Clinical characteristics

**Table 1** summarizes the clinical and metabolic variables of newly diagnosed T2DM patients as compared to healthy controls. There were significant differences in weight (p = 0.031), body mass index (BMI) (p = 0.005), HbA1c (p < 0.001), blood glucose (p < 0.001), C-reactive protein (CRP) (p < 0.001), triglycerides (TG) (p < 0.001), high-density lipoprotein cholesterol (HDLc) (p = 0.002), TG/HDL index (p < 0.001) and ApoB (p = 0.044) between newly diagnosed T2DM patients and control subjects. All variables, except HDLc, were higher in the T2DM group. Age and sex distribution between groups was similar.

**Table 1:** Data are expressed as mean ± SD.

	Control (1)	T2DM onset (2)	T2DM-1year (3)	T2DM-1year HbA1c≥6.5 (4)	T2DM-1year HbA1c<6.5 (5)	*P* (2 *vs* 1)	*P* (3 *vs* 2)	*P* (4 vs 2)	*P* (5 vs 2)
Age (years)	53.58 ± 4.81	56.52 ± 9.41	57.52 ± 9.41	57.00 ± 6.16	55.67 ± 10.09	0.155			
Sex (M/F) (%)	75/25	75.9/24.1	75.9/24.1	100/0	71.4/28.6				
Weight (kg)	78.62 ± 12.67	92.71 ± 19.02	89.56± 14.71	83.63 ± 15.48	91.12 ± 14.45	0.031	0.038	0.435	0.008
BMI (Kg/m^2^)	27.26 ± 2.59	33.12 ± 6.81	31.98 ± 5.60	29.23 ± 5.10	32.55 ± 5.65	0.005	0.049	0.422	0.039
HbA1c (%)	5.37 ± 0.23	11.91 ± 2.11	6.23 ± 0.70	7.23 ± 0.51	5.95 ± 0.43	<0.001	<0.001	0.008	<0.001
Glucose (mg/dL)	87.00 ± 10.95	156.12 ± 60.10	117.98±19.46	130.68 ± 16.05	114.36 ± 19.12	<0.001	0.010	0.366	0.004
CRP (mg/L)	1.48 ± 1.02	9.02 ± 7.52	3.96± 3.77	5.35 ± 4.73	3.57 ± 3.49	<0.001	0.005	0.542	0.006
TC (mg/dL)	188.28 ± 39.20	187.67 ± 39.01	179.87 ± 49.74	247.03 ± 32.06	160.68 ± 34.92	0.819	0.606	0.065	0.042
TG (mg/dL)	78.18 ± 34.63	152.19 ± 66.55	160.87 ± 118.98	282.91 ± 187.47	126.01 ± 62.00	<0.001	0.489	0.071	0.127
HDLc (mg/dL)	54.24 ± 12.76	39.16 ± 8.37	43.67 ± 7.56	42.44 ± 9.45	44.03 ± 7.17	0.002	0.004	0.593	0.003
LDLc (mg/dL)	118.28 ± 33.22	119.18 ± 32.60	106.03 ± 39.15	156.60 ± 28.79	91.59 ± 28.26	0.586	0.086	0.294	0.008
ApoB (mg/dL)	0.89 ± 0.28	1.06 ± 0.26	0.98± 0.33	1.34 ± 0.27	0.85 ± 0.24	0.044	0.150	0.138	0.011
TG/HDLc ratio	1.56 ± 0.93	4.21 ± 2.60	3.87 ± 3.13	7.05 ± 5.04	2.96 ± 1.57	<0.001	0.749	0.114	0.042

Comparison between groups was performed by Mann-Whitney U test and Paired Samples T-Test. BMI, Body Mass Index; HbA1c, glycated hemoglobin; CRP, C reactive protein; TC, Total cholesterol; TG, triglycerides; HDLc, High density lipoprotein cholesterol; LDLc , low density lipoprotein cholesterol; ApoB, apolipoprotein B, T2DM, type 2 diabetes mellitus.

### Glycemic control optimization normalizes the main metabolic and lipidic variables at a 12-month follow-up

As summarized in **Table 1**, the main variables that were optimized after 12 months of T2DM metabolic control were weight, BMI, HbA1c levels, glucose, HDLc and CRP.

From the total 29 T2DM patients, 23 reached a strict glycemic control (HbA1c ≤ 6.5) while only 6 remained in less strict glycemic control (HbA1c > 6.5). As shown in **Table 1**, most metabolic variables including weight, BMI, glucose, CRP, TC, LDLc, ApoB, TG/HDLc index and HDLc only significantly improved in the group reaching a strict glycemic control after 1 year.

### Newly diagnosed T2DM patients showed increased sLRP1 and decreased ANP plasma levels compared to controls

**Figure 1:**
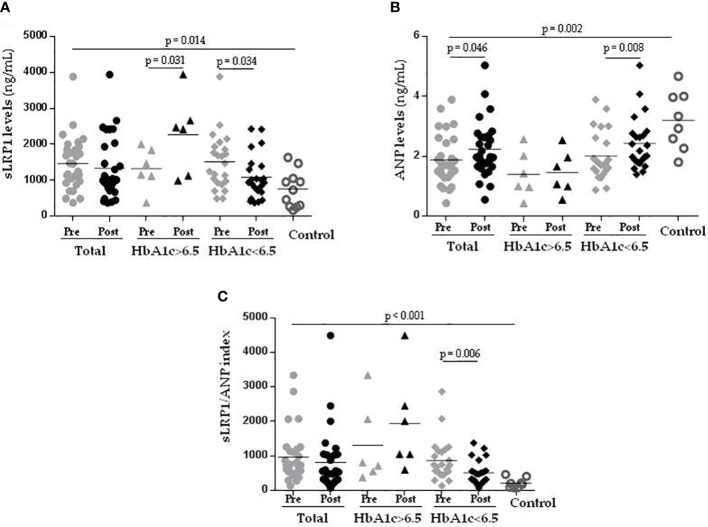
Newly diagnosed T2DM patients have increased sLRP1 and reduced ANP plasma levels that are normalized after 1 year of optimal glycemic control. Graph showing circulating levels of sLRP1 **(A)**, ANP **(B)** and sLRP1/ANP ratio **(C)** in control subjects (n = 12), Total (n = 27) (Pre =T2DM onset), (Post = T2DM-1year), T2DM-1year HbA1c>6.5 (n = 6) (Pre =T2DM onset), (Post = T2DM-1year), and T2DM-1year HbA1c ≤ 6.5 (n = 21) (Pre =T2DM onset), (Post = T2DM-1year). The horizontal line gives the mean of values from the same group. Comparisons between groups were analyzed with the Wilcoxon-test. T2DM, type 2 diabetes mellitus.

As shown in **Figure 1**, circulating sLRP1 concentrations were significantly higher in the T2DM group at onset than in the control group (p = 0.014), while those of ANP were lower (p = 0.002). Therefore, the sLRP1/ANP ratio was much higher in new-onset T2DM patients than in healthy controls (p < 0.001). Possible correlations between sLRP1, ANP, sLRP1/ANP and clinical and metabolic variables was analyzed in T2DM patients. sLRP1 directly correlated with circulating TGs (*r* ^2 ^= 0.475; p = 0.009), TG/HDLc index (*r* ^2 ^= 0.445; p = 0.015) and ApoB (*r* ^2 ^= 0.374; p = 0.046). ANP inversely correlated with circulating ApoB (*r* ^2^=-0.374; p = 0.046), CT (*r* ^2^ = -0.378; p = 0.043) and LDLc (*r* ^2^ = -0.367; p = 0.05). The ratio sLRP1/ANP directly correlated with ApoB (*r* ^2 ^= 0.506; p = 0.005), TG/HDLc index (*r* ^2 ^= 0.424; p = 0.022), LDLc (*r* ^2 ^= 0.375; p = 0.045), TGs (*r* ^2 ^= 0.392; p = 0.035), and TC (*r* ^2 ^= 0.400; p = 0.032).

### Metabolic optimization normalizes circulating levels of sLRP1 and ANP, and sLRP1/ANP ratio at a 12-month follow-up

To analyze the evolution of circulating levels of sLRP1 and ANP, and of the sLRP1/ANP ratio according to the glycemic control, the changes in these variables from T2DM onset (Pre) to T2DM-1-year (Post) were analyzed in strict glycemic control (HbA1c ≤ 6.5%) *versus* less strict glycemic control (HbA1c > 6.5%) patients (**Figure 1**).

After 1 year of treatment, sLRP1 levels were significantly downregulated in T2DM patients with a strict glycemic control, but upregulated in those with less strict glycemic control (**Figure 1A**). ANP levels were upregulated after 1 year of treatment in patients reaching a strict glycemic control but not in those that maintained HbA1c > 6.5% over 1 year of treatment, in which ANP levels did not change (**Figure 1B**). Coherently, sLRP1/ANP ratio was significantly downregulated after one year of glycemic control only in those patients that reached a strict glycemic control (**Figure 1C**).

Correlations between classical and new variables changes over 1 year were analyzed by Spearman correlation analysis. ΔsLRP1 directly correlated with changes in metabolic variables such as ΔWeight (*r* ^2 ^= 0.643; p < 0.001), ΔBMI (*r* ^2 ^= 0.636; p < 0.001), ΔTG (*r* ^2 ^= 0.668; p < 0.001), ΔGlucose (*r* ^2 ^= 0.481; p = 0.011), ΔTG/HDL (*r* ^2 ^= 0.621; p = 0.001) and inversely with the change in ΔANP (*r* ^2^ = –0.402; p = 0.031). In addition, the reduction in ΔsLRP1 also directly correlated with reductions in circulating lipids including ΔTC (*r* ^2 ^= 0.673; p < 0.001), ΔLDLc (*r* ^2 ^= 0.534; p = 0.004), and ApoB (*r* ^2 ^= 0.736; p < 0.001).

Furthermore, we analyzed whether the correlation between ΔsLRP1 and other variables, like ΔTG, ΔTC, ΔLDL, ΔApoB and ΔTG/HDL, was maintained by considering possible confounding factors, such as weight or BMI (by simple linear regression). The sLRP1 correlation adjusted for weight was significant with TG (p < 0.000), TC (p = 0.001), LDL (p = 0.002), TG/HDL (p < 0.000) and ApoB (p < 0.000). The sLRP1 correlation adjusted by BMI was also significant for TG (p = 0.001), TC (p = 0.002), LDL (p = 0.004), TG/HDL (p < 0.000) and ApoB (p = 0.001). Therefore, the correlation between the reduction in sLRP1 and lipid variables was independent of the reduction in weight or BMI.

## Discussion

This study revealed for the first time that blood sLRP1 levels, which predict cardiovascular risk (9), are upregulated, while ANP levels, which inversely predict metabolic risk (21–23), are downregulated in T2DM patients at disease onset as compared to healthy controls. In addition, we showed that sLRP1 and ANP inversely evolved after 1 year of treatment.

The elevated circulating sLRP1 levels in T2DM patients found here are in line with the documented increased LRP1 levels in the epicardial fat of T2DM patients (16) and in the myocardium of diabetic rats (13), and also with the close association between blood sLRP1 levels and the extension of epicardial fat in patients with type 1 diabetes and in the general population (17, 18). On the other hand, the low levels of ANP found in newly diagnosed T2DM patients are in line with the potential of low ANP levels to predict the development of diabetes in humans (23–27).

Previous studies from our group conducted in an experimental murine model evidenced that reduced levels of cardiac LRP1 promote increased circulating ANP levels, while increased levels of LRP1 favor decreased circulating ANP levels (22). Through the control of circulating ANP levels, cardiac LRP1 modulates not only fatty acid metabolism in the liver but also whole-body metabolism (22). Results from this experimental murine model highlight the presence of a functional LRP1-ANP link between heart, liver and adipose tissue. In the present study, a crucial point is that baseline sLRP1 levels are increased, while ANP are decreased, in newly diagnosed T2DM patients (without previous treatment) as compared to control subjects. In addition, if glycemic optimization is achieved after 1 year of treatment, there is a tight and inverse correlation between decreased sLRP1 levels and increased ANP levels. Taken together, these results suggest that the link between sLRP1 and ANP, previously described by our group in an *in vivo* model, is likely present in T2DM patients. In addition, our results support the concept that sLRP1 and ANP, which are key mediators of cardiometabolic mechanisms, are connected in humans and can play a key role in the interplay between cardiac and metabolic alterations.

Results from the present study show that strict glycemic control (HbA1c ≤ 6.5%) is highly efficient in reducing sLRP1 levels and increasing ANP levels in T2DM patients to the same levels found in the control group. These results suggest that an optimal glycemic control of T2DM patients may exert beneficial effects on parameters associated to cardiovascular risk, such as LRP1 (9). Currently, there is an intense debate about the potential and mechanisms of lowering HbA1c to provide protection against cardiovascular complications of T2DM (28, 29). Over the last decade, one of the proposed markers of glycemic control has been TG/HDLc (30–32). Results from the present study evidenced a positive correlation of sLRP1 with TG and with the TG/HDLc ratio in line with previous studies by our group showing the association of circulating TGs with epicardial LRP1 levels in T2DM patients (16). Here, we also showed that the decline in sLRP1 over 1-year caused by achieving a strict glycemic control correlated with the decrease in TGs and TG/HDL. This association between sLRP1 and TG remained after adjusting by weight and BMI. At this moment, the mechanisms underlying this association remain unclear. TG/HDLc has been proposed to be associated with CVD in the context of metabolic conditions (30–32), and sLRP1 has been reported to be predictive of cardiovascular risk in a case-cohort study (9). Therefore, it seems important to ascertain the mechanisms that determine the close association between sLRP1 and TG/HDLc in order to elucidate new mechanisms that are potentially involved in increasing the cardiovascular risk of patients with diabetes.

Here, we also observed that the reduction in sLRP1 after reaching optimal glycemic control correlated with the changes in other lipid parameters, such as TC, LDL-C and ApoB100. Such correlations were previously observed in different populations, in which sLRP1 is associated with cardiovascular risk (7–9). Further studies will be required to know whether sLRP1 reduction is associated to a decrease of atherosclerosis in T2DM patients. In addition, further studies with increased numbers of participants and long-term follow-up are required to validate the potential of sLRP1 and sLRP1/ANP to predict the beneficial effects of lipidic/glycemic control in cardiac and metabolic alterations

### Strengths and limitations

The main limitation of this study is the reduced number of participants due to the difficulty to recruit newly diagnosed untreated T2DM patients. One remarkable strength is the homogeneity of the newly diagnosed T2DM patients included in this study in terms of lack of previous hypoglycemic, lipid lowering or anti-inflammatory treatments.

## Conclusion

**Figure 2:**
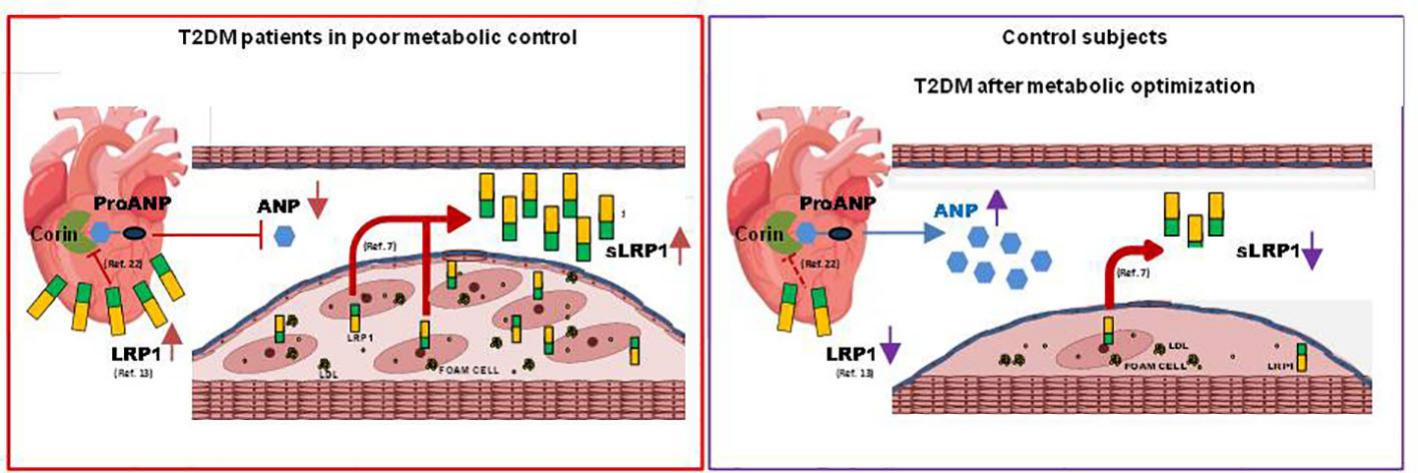
This graphical abstract combines previous findings from our group in a murine model of prediabetes with results obtained in the current study in T2DM patients. In the murine model, the reduction of cardiomyocyte LRP1 levels causes the increase of corin enzyme activity and ANP release (22). In addition, LRP1 levels are increased in the myocardium of diabetic hearts (13) and circulating sLRP1 levels correlate with atherosclerosis in humans (7). The main findings of the present study are that circulating levels of sLRP1 are increased while those of ANP are decreased in newly diagnosed type 2 diabetic patients (T2DM), and that altered sLRP1 and ANP levels are normalized in the T2DM group that reached an optimal metabolic control.

Results from the present study show that the cardiac LRP1-ANP axis previously reported by our group in an experimental murine model of prediabetes is likely working in T2DM patients. The high levels of sLRP1 in the cardiovascular system of T2DM patients could lead to low circulating levels of ANP, explaining the high sLRP1/ANP ratio found in these patients as compared to healthy controls (summarized in **Figure 2**). Altered sLRP1 and ANP levels are normalized in the T2DM group that reached an optimal metabolic control. Therefore, we propose that sLRP1/ANP can be a potential marker of the cardiovascular benefits of glycemic control in T2DM patients. Further studies are required to know whether sLRP1/ANP index might improve the predictive value of other biomarkers in terms of cardiovascular and metabolic outcomes in T2DM patients.

## Data availability statement

The raw data supporting the conclusions of this article will be made available by the authors, without undue reservation.

## Ethics statement

The studies involving human participants were reviewed and approved by Ethics Committee of the Hospital de SantPau (protocol IIBSP-REL-2017-27). The patients/participants provided their written informed consent to participate in this study.

## Author contributions

EG and PG performed ELISA assays, organized the database and did the statistical analysis. IM, JR and PG researched clinical data. AB-A collected data and contributed to the discussion. CR contributed to generation of the database. LC performed ELISA assays and collected data. JJ, SB and JS-Q designed the study and contributed to the generation of data and to discussion. XG-M and DV contributed to discussion of data. AP designed the study and wrote the manuscript. VLl-C designed the study, researched laboratory data and wrote the manuscript. All authors contributed to the article and approved the submitted version.
